# Glucose Enhances Pro-Tumorigenic Functions of Mammary Adipose-Derived Mesenchymal Stromal/Stem Cells on Breast Cancer Cell Lines

**DOI:** 10.3390/cancers14215421

**Published:** 2022-11-03

**Authors:** Maria Rosaria Ambrosio, Giusy Mosca, Teresa Migliaccio, Domenico Liguoro, Gisella Nele, Fabrizio Schonauer, Francesco D’Andrea, Federica Liotti, Nella Prevete, Rosa Marina Melillo, Carla Reale, Concetta Ambrosino, Claudia Miele, Francesco Beguinot, Vittoria D’Esposito, Pietro Formisano

**Affiliations:** 1 URT “Genomic of Diabetes”, Institute for Experimental Endocrinology and Oncology “G. Salvatore”, National Research Council (IEOS-CNR), Via Pansini 5, 80131 Naples, Italy; 2 Department of Translational Medicine, University of Naples “Federico II”, Via Pansini 5, 80131 Naples, Italy; 3 Department of Public Health, University of Naples “Federico II”, Via Pansini 5, 80131 Naples, Italy; 4 Department of Molecular Medicine and Medical Biotechnology, University of Naples “Federico II”, Via Pansini 5, 80131 Naples, Italy; 5 Institute of Genetic Research “G. Salvatore” Biogem, Via Camporeale, 83031 Ariano Irpino, Italy; 6 Department of Science and Technology, University of Sannio, Via De Sanctis, 82100 Benevento, Italy

**Keywords:** breast cancer, mammary adipose tissue, mesenchymal stromal/stem cells, glucose

## Abstract

**Simple Summary:**

Metabolic alterations (i.e., high glucose levels) enhance cancer cell aggressiveness by acting on tumor and on its environment. Mammary adipose tissue-derived mesenchymal stromal/stem cells (MAT-MSCs) stand in close proximity of breast cancer (BC) cells but have a still unclear role. We investigated whether changes in glucose concentration may impact on the interaction between human MAT-MSCs and BC cells. We provided evidence that in presence of cancer cells and high glucose levels, MAT-MSCs display pro-tumorigenic functions, while cancer cells become more aggressive. These results highlight that a metabolic control helps to reduce cancer outgrowth, at least in part preserving the functions of the adipose microenvironment.

**Abstract:**

Adiposity and diabetes affect breast cancer (BC) progression. We addressed whether glucose may affect the interaction between mammary adipose tissue-derived mesenchymal stromal/stem cells (MAT-MSCs) and BC cells. Two-dimensional co-cultures and spheroids were established in 25 mM or 5.5 mM glucose (High Glucose-HG or Low Glucose-LG) by using MAT-MSCs and MCF7 or MDA-MB231 BC cells. Gene expression was measured by qPCR, while protein levels were measured by cytofluorimetry and ELISA. CD44^high^/CD24^low^ BC stem-like sub-population was quantified by cytofluorimetry. An in vivo zebrafish model was assessed by injecting spheroid-derived labeled cells. MAT-MSCs co-cultured with BC cells showed an inflammatory/senescent phenotype with increased abundance of IL-6, IL-8, VEGF and p16^INK4a^, accompanied by altered levels of CDKN2A and *LMNB1*. BC cells reduced multipotency and increased fibrotic features modulating OCT4, SOX2, NANOG, αSMA and FAP in MAT-MSCs. Of note, these co-culture-mediated changes in MAT-MSCs were partially reverted in LG. Only in HG, MAT-MSCs increased CD44^high^/CD24^low^ MCF7 sub-population and promoted their ability to form mammospheres. Injection in zebrafish embryos of HG spheroid-derived MCF7 and MAT-MSCs was followed by a significant cellular migration and caudal dissemination. Thus, MAT-MSCs enhance the aggressiveness of BC cells in a HG environment.

## 1. Introduction

Diabetes and cancer are serious diseases, whose prevalence is rapidly increasing worldwide. It has been estimated that the number of diabetic patients will reach 578 million in 2035 and 700 million in 2045 [1] and that the number of global cancer patients will be 22 million in 2032 [2]. Epidemiological and biological studies have associated type 2 diabetes (T2D) and cancer [3]. In particular, the risk of developing breast cancer (BC) is 20–28% higher in diabetic than in healthy patients [4]. Notably, BC prognosis and overall/disease-free survival are worsened by diabetes or impaired glucose tolerance [5,6].

Cancer cells establish bidirectional communication with surrounding stromal cells to reprogram the microenvironment and obtain support for their own growth and dissemination [7]. Importantly, they accumulate metabolic alterations and take advantage of selected metabolites (including glucose), also affecting the fate of normal cells within the tumor microenvironment [8].

The breast microenvironment is composed of numerous stromal cell types, including endothelial and immune cells, fibroblasts and adipocytes. Stromal cells enhance tumor progression through mutual and dynamic communication with cancer cells [9,10,11]. For instance, cancer cells can transform stromal fibroblasts into myofibroblastic phenotypes called cancer-associated fibroblasts (CAFs) and metastasis associated fibroblasts (MAFs). CAFs secrete growth factors and cytokines to sustain cancer progression; MAFs convoy cancer cells for metastasis [12]. Adipocytes contribute to sustaining tumor phenotypes by either acting as energy reservoirs for neighboring embedded cancer cells or through secretion of signaling molecules and vesicles containing proteins, lipids and nucleic acids [9,11]. Notably, adipocytes mediate, at least in part, the impact of metabolic alterations on the BC phenotype. Indeed, we have demonstrated that glucose promotes adipocyte release of IGF1, CCL5 and IL-8 to support BC cell proliferation, invasiveness and drug-resistance [13,14,15].

Nevertheless, adipose tissue (AT) is an abundant source of mesenchymal stromal/stem cells (MSCs) showing self-renewal activity, multi-lineage differentiation potential and secreting a plethora of factors with immunomodulatory, angiogenic, neurotrophic, pro- and anti-inflammatory functions [16,17,18]. MSCs are largely modified by external stimuli. For instance, cytokines secreted by BC cells seem to play a role in the transformation of bone marrow-MSCs in CAF [19]. Moreover, exogenous factors, such as metabolic and stress-related insults may trigger a senescent phenotype in MSCs. Senescent cells display changes in gene expression and a characteristic secretory pattern called the senescence-associated secretory phenotype (SASP), which includes multiple cytokines, growth factors, proteases and matrix metalloproteinases. In most studies, the SASP was reported to sustain cancer cells [20,21]. Thus, by switching on multiple, but still unclear mechanisms, MSCs may favor tumor growth and progression. However, different studies indicate MSCs as vectors for consolidated and innovative therapies [9,22,23,24,25].

Here, we have attempted to clarify whether glucose may interfere with the dialogue between mammary adipose-derived MSCs (MAT-MSCs) and BC cells.

## 2. Materials and Methods

### 2.1. Isolation of MSCs from MAT

MAT biopsies were obtained from healthy women (N = 20) undergoing surgical mammary reduction, free of neoplastic, metabolic or endocrine diseases. Informed consents were collected before surgical procedure. Protocol was approved by the ethical committee of the University of Naples “Federico II” (prot. n. 138/16). Biopsies were minced and digested by collagenase solution (cat. C2139-1G, 1 mg/mL-Sigma-Aldrich, St. Louis, MO, USA) to isolate MAT-MSCs [13,16,26].

### 2.2. Cell Cultures

MAT-MSCs were cultured in DMEM-F12 (1:1). MCF7 (ER^+^, PR^+^, HER2^−^) and MDA-MB231 (ER^−^, PR^−^, HER2^−^) human BC cell lines were cultured in DMEM 25mM glucose. Both media were supplemented with 10% FBS, 2 mM glutamine, 100 unit/mL penicillin, and 100 unit/mL streptomycin. As indicated below, co-culture experiments cells were exposed to 25 mM glucose (standard/high glucose; HG), resembling hyperglycemia in humans, or to 5.5 mM glucose (Low Glucose; LG), resembling normoglycemia. Cultures were maintained in a humidified atmosphere of 95% air and 5% CO_2_ at 37 °C. Media, sera and antibiotics were obtained from Lonza (Basel, Switzerland). Cells were routinely screened for mycoplasma contamination.

### 2.3. Adipogenic and Osteogenic Differentiation of MAT-MSCs

Adipocyte differentiation of MAT-MSCs was obtained by the alternation (every three days, two times) of an adipocyte differentiation Induction Mix (850 nM insulin, 10 μM dexamethasone, 0.5 mM 3-IsoButyl-1-MethylXanthine, 33 μM biotin, 17 μM pantothenate and 1 μM rosiglitazone) and an adipocyte differentiation maintaining mix (850 nM insulin and 1 μM rosiglitazone). Then, the cells were stimulated (every two days, two times) with 1 μM rosiglitazone. All the process was carried out in complete culture medium. Adipocyte differentiation was reached within 17 days and lipid accumulation determined by oil red O staining [27]. Osteogenic differentiation of MAT-MSCs was reached within 21 days and mineralization foci detected by alizarin red S [16]. Images of stained monolayers were taken using the Olympus DP20 microscope digital camera system (Olympus Corporation, Tokyo, Japan). All chemicals were obtained from Sigma-Aldrich (St. Louis, MO, USA).

### 2.4. 2D-Cultures

MAT-MSCs (2.5 × 10^4^ cells) were seeded in the bottom chamber of a transwell-6 culture system (cat. 353090, 0.4 μm pore size, Costar, MA, USA) while MCF7 or MDA-MB231 were plated (3.5 × 10^4^ cells) in the upper chamber. Cells were co-cultured in the presence of HGor LG. In parallel, BC cells and MAT-MSCs were monocultured in HG or LG medium. After 72 h, conditioned media (CM) were collected, and cells were harvested for RNA extraction or cytofluorimetric analysis.

### 2.5. 3D-Cultures

Mammosphere-forming assay: MCF7 cells were plated in ultra-low attachment 96-wells (cat. 7007, Corning, NY, USA) with or without MAT-MSCs (ratio 4:1) in HG or LG medium supplemented with 5% MammoCult^TM^ proliferation supplement (cat. 05622, STEMCELL Technologies, Vancouver, BC, Canada). After 10 days, mammosphere number was quantified (number of formed spheres/number of wells containing cells × 100) [28]. In parallel, mammosphere diameter was measured by a software associated to the Olympus DP20 microscope digital camera system.

Spheroid formation: MCF7 (4 × 10^5^) were plated in ultra-low attachment 100 mm dish (cat. 3262, Corning, NY, USA) with or without MAT-MSCs (1 × 10^5^) in HG or LG medium supplemented with 5% FBS. After 72 h, spheroids were mechanically disaggregated to obtain cells for cytofluorimetric analysis or zebrafish injection.

### 2.6. Cytofluorimetric Analysis

Immunophenotypic characterization of MAT-MSCs: MAT-MSCs were incubated with PE-anti-CD73 (Cat. 550257), FITC-anti-CD90 (Cat. 555595) and APC-Cy7-anti-CD45 antibodies (Cat. 641399) as well as dye/isotype-matched antibodies (all from BD Biosciences, San Diego, CA, USA), following manufacturer’s instructions.

Quantification of CD44^high^/CD24^low^ BC cell sub-population: MCF7 were incubated with APC-anti-CD44 (cat. 130-113-338) and PE-anti-CD24 (cat. 130-112-656) as well as dye/isotype control matched antibodies (cat. 130-112-656, all from Miltenyi Biotec, Bergisch Gladbach, Germany), following manufacturer’s instructions.

Protein levels in 3D-cultures: Cell membranes were permeabilizated by using the Cytofix/Cytoperm kit (cat. 554714, BD Biosciences) before incubation (4C, 30 min) with specific or isotype control antibodies. Positivity to cytokeratin (FITC-anti-cytokeratin; cat. 130-112-743, Miltenyi Biotec) was used to discriminate MCF7 from MAT-MSCs. PE-anti-OCT4 was from Cell Signaling (cat. 2750S, Danvers, MA, USA), anti-Nanog was from Santa Cruz Biotechnology (cat. sc-374103, Dallas, TX, USA), secondary anti-mouse PE antibody used to conjugate Nanog antibody was from R&D System (cat. F0102B, Minneapolis, MN, USA), anti-αSMA was from Dako (cat. M0851, Carpinteria, CA, USA), secondary anti-mouse PE antibody used to conjugate anti-αSMA antibody was from Miltenyi Biotec (cat. 130-095-908), APC-anti-FAP was from R&D System (cat. FAB3715A), APC-anti-p16 was from Miltenyi Biotec (cat. 130-116-138). Samples were processed using a BD LSR Fortessa or using a FACSCalibur (BD Biosciences) and analyzed by using BD FACS Diva software or Cell Quest software. 10^4^ events for each sample were acquired in all analyses.

### 2.7. Multiplex ELISA Assay

CM from co-cultured and monocultured MAT-MSCs, MCF7 and MDA-MB231 were screened for concentrations of PDGF, IL-1ra, IL-1b, IL-2, IL-3, IL-4, IL-5, IL-6, IL-7, IL-8, IL-9, IL-10, IL-12, IL-13, IL-15, IL-17, eotaxin, FGF, G-CSF, GM-CSF, IFN-γ, MCP1, MIP-1α, MIP-1β, RANTES/CCL5, TNFα and VEGF using the bio-plex multiplex human cytokine and growth factor kits (cat. M500KCAF0Y, Bio-Rad, Hercules, CA, USA) according to the manufacturer’s protocol [26].

### 2.8. RNA Isolation and Analysis

Total RNA was isolated using TRIzol solution (cat. 15596026, Life Technologies, Carlsbad, CA, USA) according to the manufacturer’s instructions. RNA samples were quantified by measuring the absorbance at 260 nm and 280 nm (NanoDrop spectrophotometer, Life Technologies). RNA integrity was analyzed by using the digital electrophoresis system Experion with the “RNA StdSens Kit” (Biorad), following the manufacturer’s instructions. Run and result analyses were performed using the Experion software. RNA quality indicator (RQI) value ≥ 9 was considered good for the further analysis.

### 2.9. RT-PCR

RNA samples were reverse-transcribed using SuperScript III Reverse Transcriptase with oligo dT primers (cat. 18080-044, Life Technologies) according to the manufacturer’s instructions. To check the amplifiable template RNA/cDNA, RT-PCR amplification of housekeeping genes was performed. Amplification reactions were set up using AmpliTaq Gold (cat. N8080247, Life Technologies) and specific primer pairs, designed by Oligo 4.0 (Supplementary Table S1).

### 2.10. Quantitative Real-Time RT-PCR (qPCR)

qPCR was performed using an iTaq Universal SYBR Green Supermix (cat. 1725124, Biorad), according to the manufacturer’s instructions for the CFX Connect Real-Time system (Biorad). Relative quantification of gene expression was measured by using 2^−ΔΔCt^ method. Expression levels were normalized for the reference sample using peptidylprolyl isomerase A (*PPIA*) as housekeeping gene.

### 2.11. In Vivo Zebrafish Model

*Cell culture and labeling*: MCF7 and MAT-MSCs from 3 different donors were labeled with red cell trackers CM-DiI (cat. C7000, Thermo Fisher Scientific, Waltham, MA, USA) and BioTraker 400 Blu (cat. SCT109, Millipore, MA, USA), respectively—according to manufacturer’s instructions—before spheroid formation. After 72 h, spheroids were trypsinized, washed and resuspended in PBS/EDTA for zebrafish xenotransplantation.

*Zebrafish husbandry and xenotransplantation*: Animal experiments were in accordance with the European Council Directive 2010/63/EU and approved by the Biogem s.c.ar.l. internal ethics committee. Animal care was in accordance with institution guidelines. *Tg(fli1:EGFP)* zebrafish line, with green fluorescent vessels was raised, maintained and paired under standard conditions. Zebrafish eggs were obtained from natural spawning and maintained at 28 °C for 48 h in E3 medium (5 mM NaCl, 0.17 mM KCl, 0.33 mM CaCl_2_, 0.33 mM MgSO_4_). Two days post-fertilization (dpf), embryos were dechorionated and anesthetized with 0.04% of tricaine (Sigma Aldrich, St. Louis, MO, USA) before cell microinjection. Approximately 100–200 cells/embryo were injected in the perivitelline space of each embryo using a pneumatic PicoPump PV830 injector (World Precision Instruments, Sarasota, FL, USA) equipped with an injection borosilicate glass needle (Sutter Instruments, Novato, CA, USA). Following transplantation (0 h post injection), larvae with correct engraftment in the yolk sac were selected under Leica M205 FA fluorescence stereo microscope (Leica, Mycrosystems, Wetzlar, Germany) for further analysis and kept at 34 °C for 72 h. Embryos injected with same volume of PBS/EDTA were defined as control embryos.

*Imaging*: Zebrafish larvae were anesthetized and evaluated at 0 and 72 h post-injection by fluorescence stereo microscope. Different filters were selected for fluorescence imaging and captured with a Leica DFC450 C camera. Images of embryos at different stages of each experimental group were analyzed with ImageJ software (National Institutes of Health, Rockville, MD, USA).

### 2.12. Statistical Analysis

Statistical analyses were performed using GraphPad Prism 7.0 software (GraphPad Software Inc., La Jolla, CA, USA). Kruskal Wallis and Friedman tests followed by Dunn’s correction were applied for multiple comparisons; Wilcoxon and Mann–Whitney U tests were used for pairwise comparisons, as indicated in figure/table legends. Percentages were compared by chi-square with Fisher’s exact test. *p*-value < 0.05 was considered statistically significant. Sample sizes were determined based on the means and variations of previous pilot experiments. Hence, no statistical power analysis was used. Replicates for all the experiments were from different samples isolated from different human specimens, as indicated in figure legends.

## 3. Results

### 3.1. Characterization of MAT-MSCs

**Figure 1:**
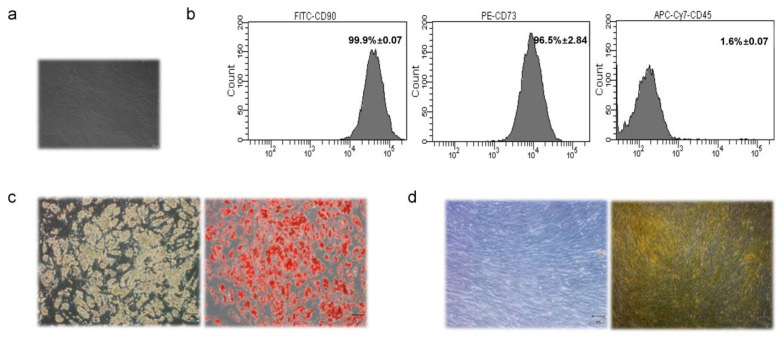
**Characterization of MSCs isolated from Mammary Adipose Tissue (MAT-MSCs)**. (**a**) Representative microscopic image (magnification 10×) of MAT-MSCs. (**b**) Representative dot plots from FACS analysis of MAT-MSCs stained for FITC-anti-CD90, PE-anti-CD73, APC-Cy7-anti-CD45 antigens. (**c**) Representative microscopic images (magnification 10×) of MAT-MSCs differentiated towards the adipogenic lineage, stained (**right**) and not (**left**) with oil red O for lipid accumulation detection. (**d**) Representative microscopic images (magnification 10×) of MAT-MSCs differentiated towards osteogenic lineage, stained (**right**) and not (**left**) with alizarin red S for calcium accumulation detection.

MSCs were isolated from MAT biopsies (N = 20) of women undergoing surgical mammary reduction (Figure 1a). Women were aged 37.4 ± 13.7, with normal glucose levels and a BMI of 27.7 ± 3.7 (Supplementary Table S2). Based on anthropometric and biochemical data, no evidence of donor sub-groups was noted (Supplementary Figure S1). MAT-MSCs expressed mesenchymal progenitor cell surface antigens CD90 (99.9% ± 0.07) and CD73 (96.5% ± 2.84) while not expressing the hematopoietic marker CD45 (1.6% ± 0.07) (Figure 1b). Moreover, upon specific stimuli (see Section 2), MAT-MSCs differentiated into both adipocytes (Figure 1c) and osteocyte-like cells (Figure 1d).

### 3.2. Profiling of Cytokines, Chemokines, and Growth Factors in MAT-MSCs and BC Cell Co-Cultures

To study the bi-directional communication between MAT-MSCs and BC cells, 2D-cultures were established. Conditioned media (CM) from co-cultured and monocultured MAT-MSCs, MCF7 and MDA-MB231 were collected to define the release of cytokines/chemokines and growth factors. Detectable levels of several molecules were observed in both monocultures and co-cultures (Table 1). The different MAT-MSC isolates displayed a similar secretory profile, without evidence of cell clusters (Supplementary Figure S2a). Moreover, no significant correlations were found between released factors and age, BMI, glucose, cholesterol and triglyceride blood levels of donors (Supplementary Figure S2b). MAT-MSCs secreted a high amount of IL-6, which was the most largely detected cytokine in co-cultures of MAT-MSCs with both MCF7 (421.07 ± 146.91 pg/mL) and MDA-MB231 cells (631.42 ± 286 pg/mL). High levels of IL-6 in co-cultures may derive from MAT-MSCs, since monocultured BC cells released much lower amounts of the cytokine (Table 1). Similar to IL-6, co-cultures of MAT-MSCs with both MCF7 and MDA-MB231 released high amounts of VEGF (173.44 ± 44.43 pg/mL and 115.12 ± 22.21 pg/mL, respectively). Finally, MSCs + MDA-MB231 co-cultures displayed high concentrations of IL-8 (47 ± 17.51 pg/mL), also largely secreted by MDA-MB231 monocultures (20.45 ± 8.71 pg/mL), while barely detected in CM from MAT-MSCs, MCF7 and MSCs + MCF7.

Thus, co-culture media, compared to MAT-MSCs and/or BC cell monoculture media had higher levels of IL-6, VEGF and IL-8; proteins involved in SASP; CAF functions and cancer aggressiveness [20,29,30,31].

### 3.3. Evaluating the Role of Glucose on BC Cell-Induced Phenotype of MAT-MSCs

**Figure 2:**
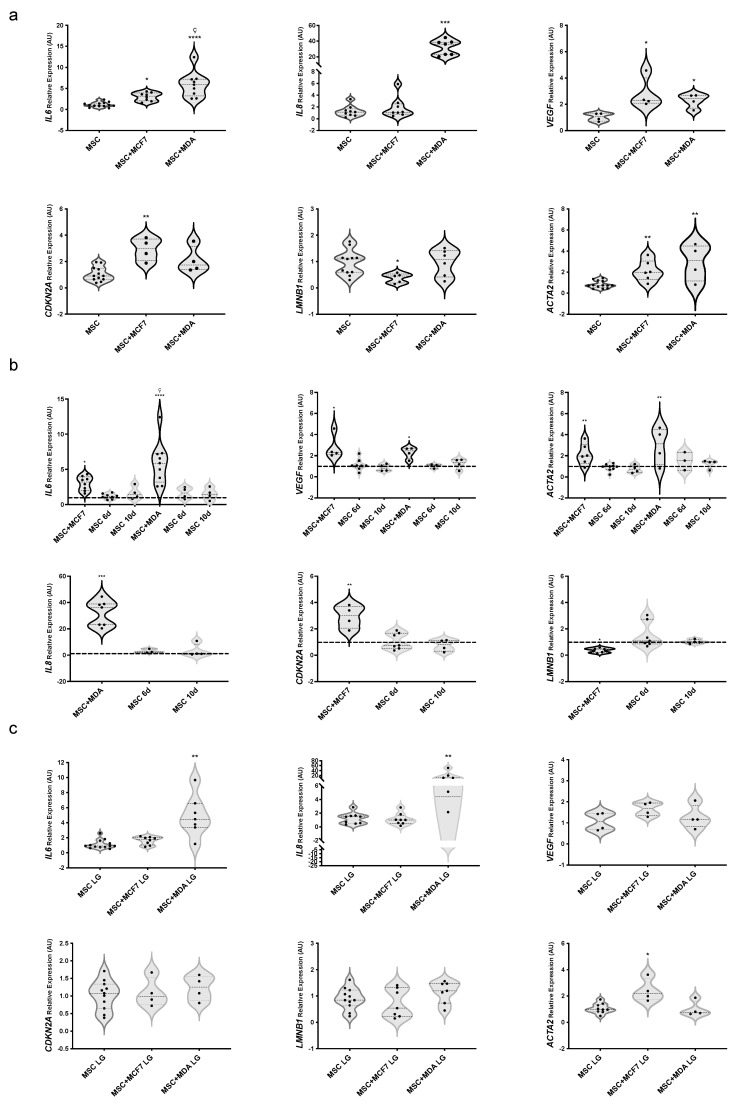
**Effect of glucose on MAT**-**MSCs phenotype in 2D**-**cultures with BC cells.** MAT-MSCs were co-cultured—and not—with MCF7 or MDA-MB231 for 72 h in (**a**,**b**) standard/high glucose (25 mM glucose; MSC) or (**c**) low glucose concentration (5.5 mM; MSC LG). After 72 h, BC cells were removed while MAT-MSCs were grown for another 3 (MSC 6d) or 7 days (MSC 10d). mRNA expression levels of senescence/fibrosis markers (*IL6, IL8, VEGF, CDKN2A, LMNB1, ACTA2*) were determined by qPCR (see Section 2 and Supplementary Table S1. Data were normalized on the peptidyl-prolyl cis-trans isomerase A (*PPIA*) gene as per internal standard. Results were represented as: violin plot of 7–9 (**a**,**c**) or 3–5 (**b**) independent triplicate experiments showing mRNA levels of *IL6*, *IL8*, *VEGF*, *CDKNA2*, *LMNB1* and *ACTA2* in MAT-MSCs co-cultured with BC cells as relative expression (2^−ΔΔCt^) compared to that in monocultured MAT-MSCs (control cells). In (**b**) monocultured cells (control cells) are represented as a dotted line. Data were analyzed using non-parametric Kruskall–Wallis test followed by Dunn’s correction for multiple comparisons. For pairwise comparisons (MSC + MCF7 vs. MSC + MDA-MB231), Mann–Whitney test was assessed. * denotes statistically significant values compared with monocultured MAT-MSCs (* adjp < 0.05; ** adjp < 0.01; *** adjp < 0.001, **** adjp < 0.0001). ^ç^ denotes statistically significant values compared with MSC + MCF7 (^ç^ adjp < 0.05).

Next, we analyzed mRNA levels of *IL6*, *IL8*, and *VEGF* together with established markers of cell senescence *CDKN2A* and *LMNB1*, in MAT-MSCs. *CDKN2A* encodes p16^INK4a^, a protein elevated in cultured senescent cells, while *LMNB1* encodes lamin B1, whose reduction is considered a senescence-associated biomarker [32,33]. As shown in Figure 2a, we found a 3.2- and 2.3-fold increase in *IL6* and *VEGF*—while not of *IL8*—mRNA levels in MAT-MSCs co-cultured with MCF7 (Figure 2a; adjp < 0.05) compared to control cells, represented by MAT-MSCs monocultures. Furthermore, we observed that co-cultures with MDA-MB231 induced *IL6*, *IL8* and *VEGF* mRNAs in MAT-MSCs by 5.9-, 36.3- and 2.4-fold, respectively (Figure 2a; adjp < 0.001). Of note, co-cultures with MCF7, while not with MDA-MB231, modified the expression levels of *CDKN2A* (3-fold increase) and *LMNB1* (about 50% reduction) in MAT-MSCs (Figure 2a; adjp < 0.05). Then, we measured mRNA levels of *ACTA2*, encoding α-SMA, a marker of myofibroblast trans-differentiation, and observed that both MCF7 and MDA-MB231 promoted its expression in MAT-MSCs by 2- and 3-fold, respectively (Figure 2a; adjp < 0.01). No correlation between gene expression profiles and antropometric and biochemical data of the donors was found in MAT-MSCs.

Interestingly, we found that BC modulation of senescence and trans-differentiation markers in MAT-MSCs was not maintained for 10 days upon the end of co-culture (i.e., the removal of BC cells) (Figure 2b).

Next, we investigated the impact of glucose lowering (25 mM to 5.5 mM; HG → LG) onto the MAT-MSCs phenotype acquired upon co-culture with BC cells. We previously demonstrated that glucose did not modify the MAT-MSC proliferation rate, while increasing BC cell proliferation [13,16]. Here, we found that glucose lowering *per sè* did not modulate *IL8*, *VEGF*, *CDKN2A* and *LMNB1* mRNA levels while it promoted *IL6* and *ACTA2* expression (Supplementary Figure S3; adjp < 0.01). Co-culturing MAT-MSCs with MCF7 in LG still induced *ACTA2* mRNA expression (Figure 2c; adjp < 0.05) while—at variance with HG (Figure 2a)—it did not affect *IL6*, *VEGF*, *CDKN2A* and *LMNB1* mRNA levels (Figure 2c).

In parallel, in LG, MDA-MB231 still upregulated *IL6* and *IL8* but lost the ability to increase mRNA levels of *VEGF* and *ACTA2* in MAT-MSCs (Figure 2c). Thus, glucose lowering reverted, at least in part, the acquisition of senescent/fibrotic phenotype induced by BC cells in MAT-MSCs.

In addition, in these co-culture systems, neither glucose nor MAT-MSCs modulated expression levels of inflammatory and senescence-associated markers in MCF7 (Supplementary Figure S4) and in MDA-MB231 (Supplementary Figure S5).

**Figure 3:**
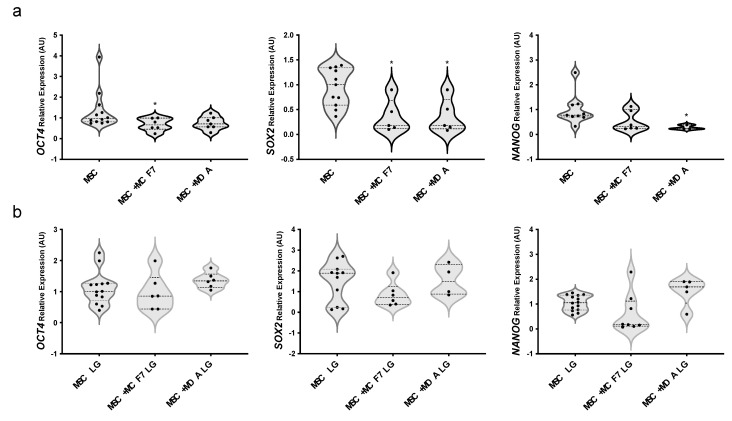
**Effect of glucose on MAT**-**MSCs multipotency in 2D cultures with BC cells.** MAT-MSCs were co-cultured—and not—with MCF7 or MDA-MB231 BC cells in (**a**) standard/high glucose (25 mM glucose; MSC) or (**b**) low glucose concentration (5.5 mM; MSC LG). After for 72 h, mRNA expression levels of multipotency markers (*OCT4, SOX2, NANOG*) were determined by qPCR (see Methods and Supplementary Table S1). Data were normalized on *PPIA* gene as internal standard. Results were represented as violin plot of 7–9 independent triplicate experiments showing mRNA levels of *OCT4, SOX2* and *NANOG* in MAT-MSCs co-cultured with BC cells as relative expression (2^−ΔΔCt^) compared to that in mono-cultured MAT-MSCs. Data were analyzed using non-parametric Kruskall–Wallis test followed by Dunn’s correction for multiple comparisons. * denotes statistically significant values compared with MSC (* adjp < 0.05).

Notably, we also observed that co-cultures with BC cells significantly reduced mRNA levels of multipotency markers *OCT4* (about 30% reduction upon co-culture with MCF7), *SOX2* (about 80% reduction upon co-culture with both MCF7 and MDA-MB231) and *NANOG* (about 80% reduction upon co-culture with MDA-MB231) in MAT-MSCs (Figure 3a; adjp < 0.05). Glucose lowering did not modify the expression of multipotency-related genes in MAT-MSCs (Supplementary Figure S3b) while totally restoring cancer cell action on their expression levels (Figure 3b).

### 3.4. Evaluating the Role of Glucose on MAT-MSC-Induced Phenotype of BC Cells

**Figure 4:**
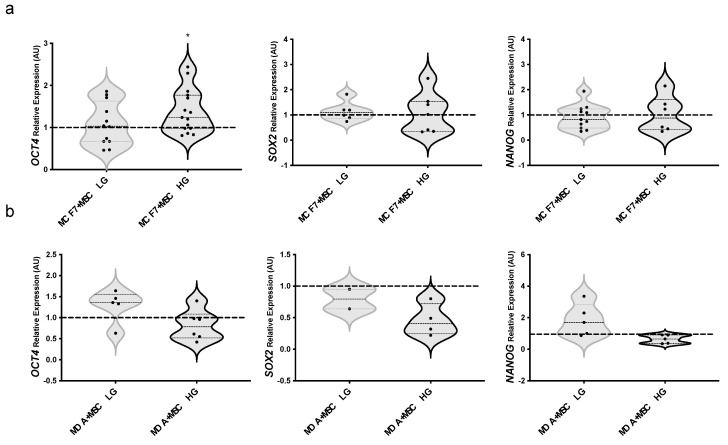
**Effect of glucose on BC cells stemness in 2D cultures with MAT-MSCs.** (**a**) MCF7 or (**b**) MDA-MB231 were co-cultured-and not—with MAT-MSCs in low glucose (5.5 mM; LG) or standard/high glucose (25 mM; HG) medium. After 72 h, mRNA expression levels of stemness genes (*OCT4, SOX2, NANOG*) were determined by qPCR (see Methods and Supplementary Table S1). Data were normalized on *PPIA* gene as internal standard. Results were represented as violin plot of 7–15 independent triplicate experiments showing mRNA levels of *OCT4, SOX2* and *NANOG* in (**a**) MCF7 + MSC LG/HG and (**b**) MDA-MB231 + MSC LG/HG as relative expression (2^−ΔΔCt^) compared to that in monocultured cells (MCF7/MDA-MB231 LG/HG; dotted line). Data were analyzed using the non-parametric Wilcoxon test for pairwise comparisons. * denotes statistically significant values (* pval < 0.05).

To investigate the effect of MAT-MSCs and the possible role of glucose to the BC cell stem-like phenotype, mRNA levels of *OCT4*, *SOX2*, and *NANOG* were measured in BC cells upon co-culture with MAT-MSCs in HG or LG. We found a significant increase of *OCT4* mRNA levels in MCF7 co-cultured with MAT-MSCs in HG while not in LG (Figure 4a; pval < 0.05) as compared to the relative monocultures. *SOX2* and *NANOG* mRNA levels remained unaltered in co-cultured MCF7 (Figure 4a). Notably, glucose did not directly affect the expression of these genes in MCF7 cells (Supplementary Figure S6). Moreover, we observed that stemness genes did not change in MDA-MB231 upon co-culture with MAT-MSCs neither in LG nor in HG (Figure 4b).

**Figure 5:**
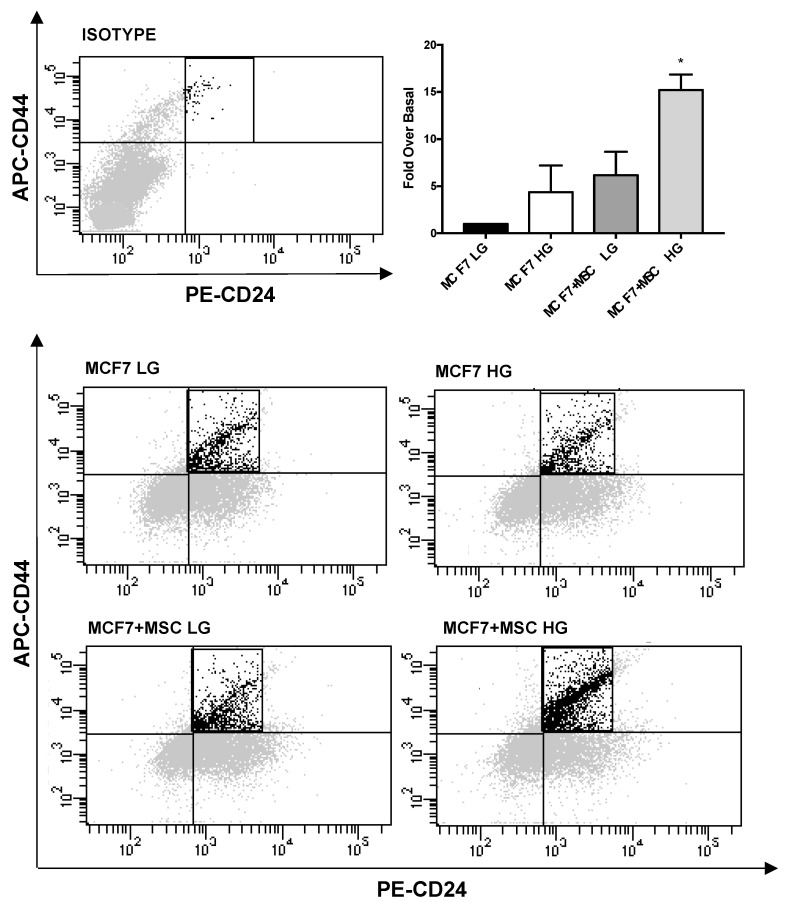
**Quantification of CD44^high^/CD24^low^ subpopulation in BC cells co-cultured with MAT-MSCs.** MCF7 were co-cultured—and not—with MAT-MSCs in LG or HG medium. After 72 h, CD44^high^/CD24^low^ stem cell subpopulation was identified in monocultured (MCF7 LG/HG) and co-cultured (MCF7 + MSC LG/HG) MCF7 by FACS analysis (See Section 2). Representative dot plots show MCF7 stained with APC-anti-CD44 and PE-anti-CD24 as well as dye/isotype matched antibodies. In the graph, the percentage of population was reported as fold over basal (MCF7 LG) and showed CD44^high^/CD24^low^ cell subpopulation as mean ± SD of 4 independent experiments. Data were analyzed using the non-parametric Friedman test followed by Dunn’s correction for multiple comparisons. * denotes statistically significant values compared with MCF7 LG (* adjp < 0.01).

Next, we quantified CD44^high^/CD24^low^ stem-like BC sub-population upon co-culture with MAT-MSCs. As shown in Figure 5, co-culturing MCF7 with MAT-MSCs in HG, while not in LG, determined a 15-fold increase of CD44^high^/CD24^low^ sub-population (Figure 5; adjp < 0.01). Thus, MAT-MSCs induced a stem-like phenotype in MCF7.

### 3.5. Stem Phenotype of MAT-MSCs and BC Cells in 3D Cultures

**Figure 6:**
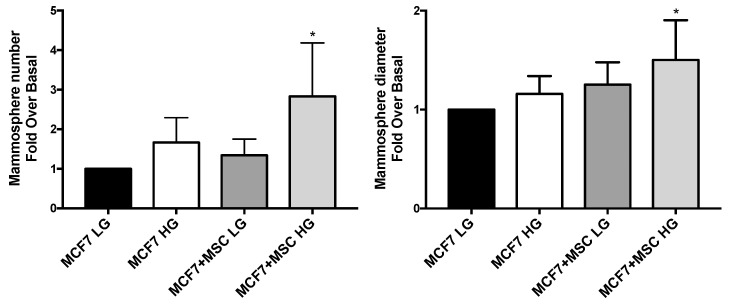
**Effect of glucose and MAT-MSCs on mammosphere formation.** Three-dimensional cultures were set up by using MCF7 and MAT-MSCs (ratio 4:1) in LG or HG concentrations. After 10 days, mammosphere number and diameter were obtained. Results were reported as fold over basal (MCF7 LG). Bars represent mean ± SD of 4–5 independent experiments showing mammosphere number and diameter in BC cells, co-cultured and not with MAT-MSCs. Data were analyzed using the non-parametric Friedman test followed by Dunn’s correction for multiple comparisons. * denotes statistically significant values compared with MCF7 LG (* adjp < 0.05).

Three-dimensional spheroids were established with MCF7 in presence and in absence of MAT-MSCs, in HG or LG. MCF7 were able to form a 2.8-fold higher number of mammospheres, also characterized by 50% increased diameter, when co-cultured with MAT-MSCs in HG, while not in LG (Figure 6; adjp < 0.05).

**Figure 7:**
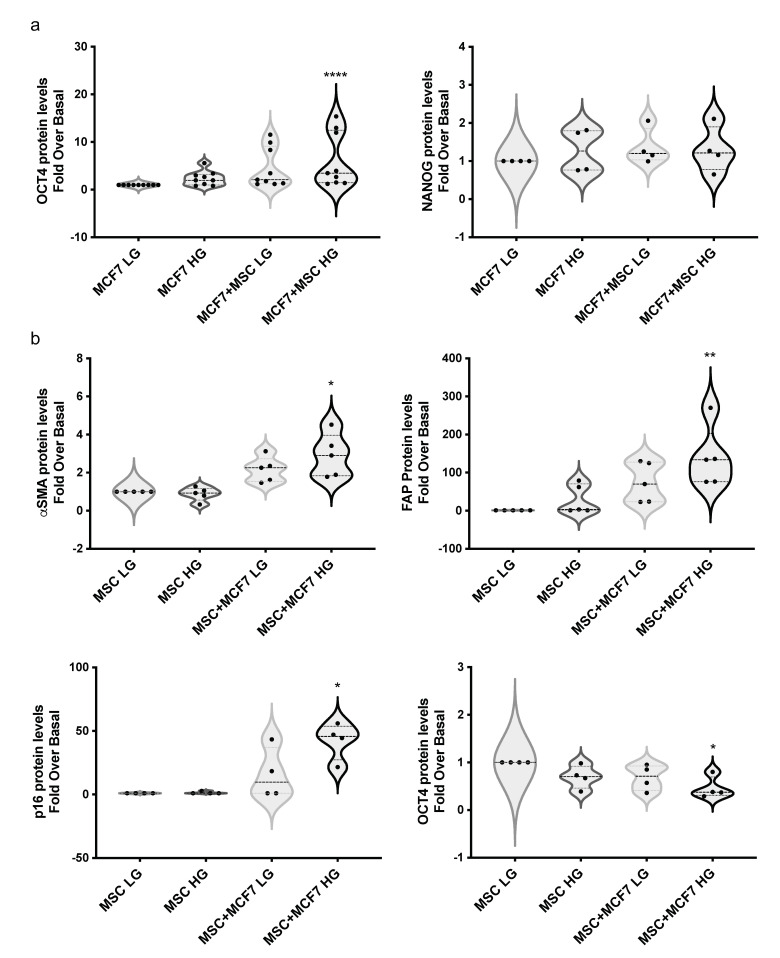
**Effect of glucose on MAT-MSCs and MCF7 crosstalk in 3D organoids.** Three-dimensional cultures were set up by using MCF7 and MAT-MSCs (ratio 4:1) in LG or HG concentrations. After 72 h, spheroids were mechanically disaggregated and cells were stained with PE-anti-OCT4, PE-anti-NANOG, PE-anti-α-SMA, APC-anti-FAP and APC-anti-p16, as well as dye/isotype matched antibodies for FACS analysis. Results were reported as violin plot of 4–9 independent experiments showing protein levels of (**a**) Oct4 and Nanog in MCF7 or (**b**) α-SMA, FAP, p16 and OCT4 in MAT-MSCs as fold over basal (MCF7 LG/MSC LG). Data were analyzed using the non-parametric Friedman test followed by Dunn’s correction for multiple comparisons. * denotes statistically significant values compared with MCF7 LG or MSC LG (* adjp < 0.05; ** adjp < 0.01, **** adjp < 0.0001).

Notably, when disaggregated, MCF7 included in spheroids with MAT-MSCs in HG displayed a 4.3-fold increase in OCT44 protein (adjp < 0.05) with no change of NANOG levels (Figure 7a, Supplementary Figure S7a). In parallel, in the same spheroids, a significant increase in α-SMA (adjp < 0.05), FAP (adjp < 0.01) and p16^INK4a^ (adjp < 0.05) proteins was observed in MAT-MSCs (Figure 7b, Supplementary Figure S7b). In addition, in these cells NANOG protein levels did not change. However, a 50% significant reduction of OCT4 (adjp < 0.05) was detected (Figure 7b, Supplementary Figure S7b).

### 3.6. Glucose Modulates Invasiveness of MAT-MSCs and MCF7 Spheroids in Zebrafish Xenograft Model

**Figure 8:**
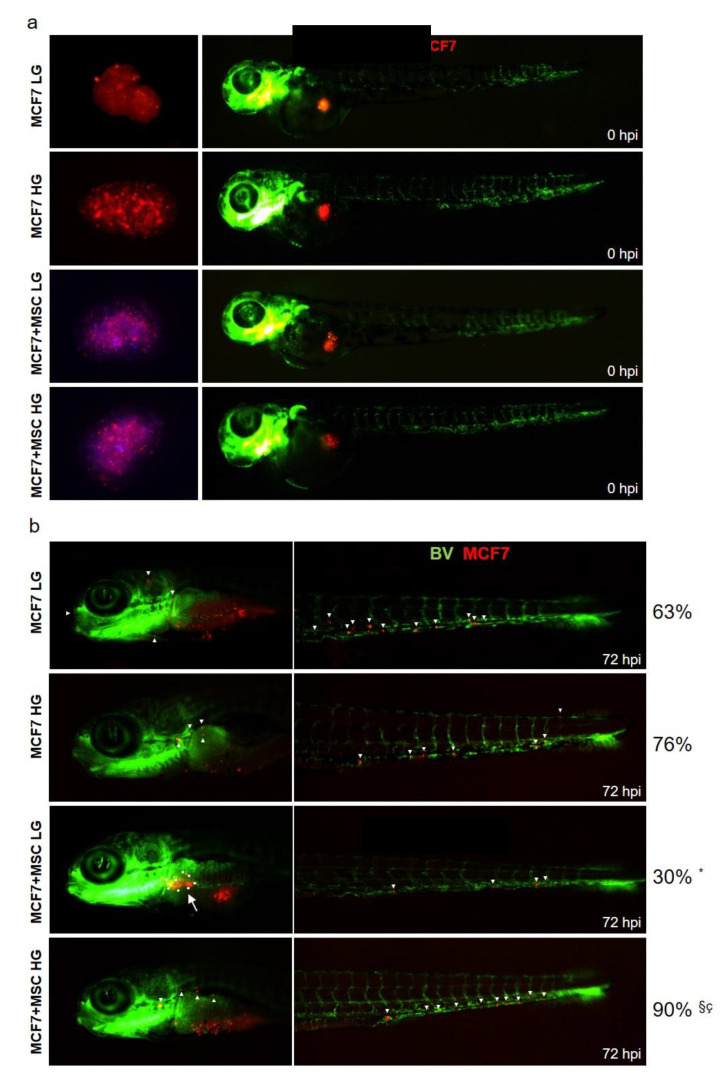
**Effect of glucose on MCF7-MAT-MSCs organoids in xenograft zebrafish models.** MCF7 (labeled with red dye), cultured in spheroids alone or with MAT-MSCs (labeled with blue dye) in LG or HG, were injected into the PVS of 48 hpf zebrafish embryos. MCF7 and MCF7 + MSCs spheroids in LG and HG ((**a**), **left**) were disaggregated, injected and visualized under fluorescence stereo microscope at time point 0 hrs post-injection ((**a**), **right**). BV = blood vessel. MCF7 dissemination in LG and HG was analyzed 72 hrs post-injection (**b**). White arrows indicate migrated MCF7 cells in zebrafish head and tail. Results have been presented as percentage of xenograft with invasive BC cells. Data were analyzed using the chi-square with Fisher’s exact test. * denotes statistically significant values compared with MCF7 LG (* pval < 0.0001). ^§^ denotes statistically significant values compared with MCF7 HG (^§^ pval < 0.01). ^ç^ denotes statistically significant values compared with MSC + MCF7 LG (^ç^ pval < 0.0001).

Finally, spheroid-derived cells were engrafted in 2-day-old zebrafish embryos (Figure 8a). Immediately after injection, cells were localized in the yolk sac (Figure 8a, red spot). At 72 h post-injection, cells were able to migrate in head and tail regions (Figure 8b, white arrows). Monocultured MCF7 in LG and HG showed a similar dissemination rate. Indeed, 63% of xenografts with invasive MCF7 in LG, and 76% with invasive cells in HG were detected (Figure 8b). Interestingly, when injected MCF7+MSCs in LG, only 30% of xenografts displayed invasive BC cells, a percentage significantly reduced as compared to xenografts with MCF7 monocultures. In this condition, the majority of zebrafish displayed a clear localized tumor mass (Figure 8b, bigger white arrow). At variance, when injected, MCF7 + MSCs in HG 90% of xenografts showed invasive cells, a percentage significantly higher compared to xenografts with MCF7 monocultures or MCF7 + MSCs in LG (Figure 8b).

Thus, glucose also modulates BC-invasive features in in vivo models.

## 4. Discussion

Obesity and type 2 diabetes are epidemic diseases strictly associated with BC risk and progression. Many systemic factors, dysregulated in obesity and T2D, may contribute to BC, including insulin, insulin-like growth factor 1 (IGF1), glucose, lipids, inflammatory cytokines, immune cells, steroids, the autonomic nervous system, adipokines and the microbiome [9,34]. These factors may have direct pro-tumorigenic effects on BC cells but may also disrupt the local tumor microenvironment (TME). For instance, high glucose levels promote the proliferation, invasion, and migration of BC cells and enhance the chemoresistance of tumors via abnormal glucose metabolism. In parallel, glucose may modify the TME, increasing the PH, the lactic acid, production of inflammatory factors, imbalance of ROS and the cellular composition/phenotype [13,35].

Within the breast, cancer cells communicate with adipocytes and their mesenchymal precursors which release signaling molecules and provide mechanical support and energy supply [9,11]. Metabolic imbalances, such as high glucose levels, may affect such communication, contributing to the association between metabolic diseases and BC progression. Previously, we demonstrated that glucose promotes adipocyte release of IGF1, CCL5 and IL-8, enhancing BC cell proliferation, invasiveness and tamoxifen resistance [13,14,15].

Here, we have shown for the first time that glucose participates in the dialogue between mammary MSCs and BC cells, which leads to a MSC CAF-like phenotype and BC cell stem-like features.

To date, the contribution of MSCs in BC progression has been investigated mainly by using murine MSCs (mMSCs), human bone marrow-derived MSCs (BM-MSCs) or human abdominal adipose tissue (AT)-derived MSCs. However, biological differences between BM-MSCs and AT-derived MSCs have been described and, in the context of AT, the functional diversity of depots is well established [16,17,36]. Indeed, although different studies reported that MSCs support BC cell growth and migration [9,23], a piece of literature assigns tumor-suppressive functions to MSCs, considering them as suitable carriers for anti-cancer drug delivery [22,37]. Recently, it has been shown that MSCs isolated from mammary AT (MAT-MSCs) of patients with BC displayed a dysregulated secretory profile and enhanced tumorigenicity compared to MAT-MSCs isolated from healthy donors, suggesting permanent changes induced by cancer cells in local MSCs [25]. Moreover, it has been reported that MAT-MSCs are largely modified by doxorubicin and paclitaxel, which influence cytokine release and potentiate the ability of MSCs to stimulate BC invasive potential in vivo [24]. Thus, local MSCs exhibit a pivotal role in BC progression and are extensively affected by external stimuli.

Here, we defined the role of MSCs in BC progression by taking advantage of MAT-MSCs obtained from a cohort of healthy women. In this population no evidence of cell clusters due to interindividual differences were found. This is consistent with other studies showing no differences in gene expression profiles in MAT-MSCs obtained from healthy donors with different ages [25]. However, a future research direction will be the deeper study of the pro/anti tumorigenic effect of MAT-MSCs obtained from well-defined groups of donors with different anthropometric/clinical data.

In this study, we closely examined the role of glucose in the communication that MAT-MSCs establish with BC cells. Two- and three-dimensional in vitro co-cultures and in vivo zebrafish embryo models were established in 25 mM or 5.5 mM glucose (HG or LG, respectively), resembling hyperglycemia or close-to-normal fasting glucose levels in humans. Through this model, we provided evidence that MSCs are largely modified by ER^+^ and TNBC cells and promote BC cell stemness in an HG environment.

Interestingly, within a screening of cytokines, chemokines and growth factors, we observed increased release of IL-6, IL-8 and VEGF in co-cultures of MAT-MSCs with BC cells. Consistently, significantly higher *IL6* and *VEGF* mRNA levels were detected when MAT-MSCs were co-cultured with MCF7 and MDA-MB231; in contrast, *IL-8* expression was increased in MAT-MSCs only upon co-culture with MDA-MB231. IL-6, IL-8 and VEGF play a crucial role in tumor microenvironment and represent key signals in the communication between adipose, cancer, endothelial and immune cells [13,28,30,31,38]. High serum levels of IL-6, IL-8 and VEGF have been associated with poor BC prognosis/overall survival [31], but also with insulin resistance and diabetic complications [13,39,40]. Hence, targeted inhibitors are actually in use or in clinical trial either for BC [38] or diabetes [39,40,41]. Notably, IL-6, IL-8 and VEGF are also considered senescence-associated secretory phenotype (SASP) factors [29]. Here, we showed their over-expression, accompanied by a de-regulation of senescence markers *LMNB1* and *CDKN2A* (the latter also at protein levels) in MAT-MSCs particularly upon co-culture with MCF7 cells, suggesting the acquisition of a senescent phenotype. In this model, although we did not find β-galactosidase (β-gal) activation, *CDKN2A* (and p16^INK4A^) increase and LMNB1 decline were detected [20,32,33]. In senescent cells their modifications appear earlier compared to β-gal, whose activity takes 7–10 days to develop upon specific senescent stimuli [32].

IL-6, IL-8 and VEGF are largely secreted also by cancer-associated fibroblasts (CAFs), the most heterogeneous population of stromal cells in the BC microenvironment. CAFs modulate cancer metastasis and influence angiogenesis, tumor mechanics, drug access and therapy responses [42,43]. Different studies reported the presence of senescent CAFs in solid tumors [20], with a more evident pro-tumorigenic role for senescent, compared to non-senescent CAFs [44,45]. Here, we have found that MAT-MSCs, cultured with BC cells, show an up-regulation of α-SMA and FAP, largely recognized markers of CAFs in BC. Notably, α-SMA expression in BC environment has been correlated with metastasis and poorer overall survival rate [46] and its expression is common to senescent and non-senescent CAFs [20]. The contemporary presence of α-SMA and FAP identifies also a new specific CAF subpopulation (referred to as CAF-S1), which promotes immunosuppression and immunotherapy resistance in BC [42,43]. FAP is still considered the most promising CAF therapeutic target. Indeed, FAP inhibition to eradicate CAFs has been associated with decreased collagen content and tumor burden, and improved immunotherapy effectiveness [47,48,49]. Our data suggest that, in presence of BC cells, MAT-MSCs acquire a CAF-like phenotype, but only in presence of ER^+^ cells they become senescent CAFs.

Interestingly, almost all markers of senescence and fibrosis were not induced by BC cells in MAT-MSCs in an LG environment. These results highlight that MAT-MSCs may transdifferentiate in CAFs and that their heterogeneity in BC could be driven by cancer subtypes. Notably, we have evidenced that fibro/senescent features acquired by MAT-MSCs upon co-culture with BC cells in HG are not stably retained. Hence, once removed from cancer cells, *IL6*, *IL8*, *VEGF*, *CDKN2A* and *LMNB1* mRNA levels in MSCs return to levels observed in MSCs without cancer cells. However, it should be reported that CAFs are characterized by high plasticity and their properties may change upon longer-term cultures [50].

The loosening of an undifferentiated state of MAT-MSCs cultured with BC cells is pointed up also by the finding that in HG co-cultures they showed a reduction of *OCT4*, *SOX2* and *NANOG*, which play a critical role for the maintenance of the multipotent/pluripotent state [51]. Few studies have investigated their functions in adult MSCs. It has been shown that these factors inhibit lineage genes by binding to the *DMT1* promoter and downregulating p16 expression [52]. Accordingly, we have detected a reduction of multipotency-related genes and an increase in p16^INK4A^. Such effects might be, at least in part, related to the increase of α-SMA. Indeed, it has been documented that α-SMA-positive BM-MSCs exhibited a fibrotic phenotype, with reduced self-renewal, adipogenic differentiation, *OCT4* and *SOX2* levels [53]. However, in LG co-cultures, we detected no difference in multipotency genes, despite increased levels of α-SMA. Another possible mechanism may be represented by the glutamine withdrawal in the culture medium. BC cells display an upregulation of glutamine metabolism [54], while on the other hand, glutamine depletion has been shown to induce OCT4 degradation in embryonic stem cells [55].

Beside controlling stem cell fate, multipotency genes display a strong association with cancer progression. Indeed, OCT4 is an essential factor for the early stages of mammalian embryogenesis, but also the major regulator of cancer stem cells (CSCs) in BC. CSCs are tumor-initiating cells with a clear role in cancer recurrence, metastasis, and drug resistance. Although different factors may contribute to CSCs generation and maintenance, the mechanisms involved are largely unknown. Moreover, patients with high OCT4 expression in BC showed poor post-progression survival [51,56,57]. Diverse studies provided evidence that MSCs, mainly of murine origin, promote the acquisition of stem-like features of BC cells, via different pathways involving cytokines (i.e., IL-6, IL-8, CCL5), growth factors and miRNAs [58,59]. Here, we have shown, for the first time, that mammary MSCs, in an HG environment, increased OCT4 levels in ER^+^ BC cells. Instead, MDA-MB231 cells did not modify stem-related genes in presence of stromal stimuli. Similar results were obtained in BT-549 triple negative breast cancer cells. At variance, in BT-474, an ER^+^ and HER2^+^ BC cell model, even though not significant, increased levels of *OCT4*, *SOX2* and *NANOG* in presence of MAT-MSCs, only in HG, were observed. These results need to be confirmed in human samples and in other BC cells, however they are in line with literature data reporting a different crosstalk between BC subtypes and the tumor microenvironment [60,61]. For instance, a recent study based on high-dimensional imaging describes how the specialized cells of the breast tumor microenvironment organize in space, how this organization varies across tumor subtypes and how this may impact clinical outcomes [60]. However, the signaling molecules that regulate the interplay between tumor environment and different BC subtypes have not yet been defined. TNBCs harbor the highest proportion of CSCs compared with other subtypes, contributing to the poor prognosis associated with this subtype [62,63]. Thus, it is possible to hypothesize that TNBC cells, which express high levels of OCT4 and hold numerous CD44^high^/CD24^low^ cells, compared to other BC subtypes, do not respond to external stimuli (i.e., MAT-MSCs, glucose) to further increase stemness features. In contrast, MCF7, a less aggressive BC model, may be more prone to capturing external stimuli, making them ancillary signals. Accordingly, in these cells, the proportion of the CD44^high^/CD24^low^ BC cell sub-population was increased in presence of MAT-MSCs in HG. Interestingly, we provided evidence also that in HG, a low number of MSCs was sufficient to expand BC mammosphere number and diameter. We found that spheroid-derived MCF7 and MAT-MSCs exhibit high OCT4 and α-SMA/FAP/p16^INK4A^ protein levels, respectively. Both spheroid-derived cells have also been injected in zebrafish embryos, which are optically transparent and permissive to the xenograft of human tumor cells. This method allows in vivo delivery of a very limited number of cancer cells, mimicking the initial stages of tumor angiogenesis and metastasis; thus, it is now considered a useful approach to study the metastatic homing and colonization [64,65,66]. Zebrafish are becoming increasingly prominent in the study of the tumor microenvironment, since they allow for the reproduction of in vivo conditions in which cancer cells normally grow, consisting of blood vessels, lymphatic vessels, stromal cells, extracellular matrices, proteins and RNAs. However, there are still many needs to address with regard to mimicking the human tumor environment [65,67]. Here, we showed that the injection of spheroid-derived MCF7 + MSCs led to higher tumor invasiveness compared to that of spheroid-derived MCF7, only in HG. Indeed, in LG the percentage of xenografts with invasive cells was significantly lower. Our model is innovative, since it is based on a xenograft generated with BC cells + MAT-MSCs spheroids. It allows for the study of tumor/MSCs crosstalk during cancer cell invasion and metastasis and may be envisioned as novel approach for drug screening toward breast cancer.

## 5. Conclusions

In high glucose environments BC cells induce profound changes in mammary MSCs, increasing the expression of inflammation, senescence and fibrosis markers, and downregulating multipotency genes. Tumor-educated MAT-MSCs, in turn, promote a CSC phenotype in ER^+^ BC cells and tumor invasiveness in an in vivo model. Glucose lowering interferes with the dangerous communication between cancer cells and MAT-MSCs, counteracting, at least in part, these pro-tumorigenic effects.

## Figures and Tables

**Table 1:** **Cytokine, chemokine and growth factor concentration (pg/mL) in conditioned media (CM) from mono-cultured and co-cultured cells.** MCF7 or MDA-MB231 were co-cultured—and not—with MAT-MSCs in standard/high glucose (25 mM glucose; MSC). After 72 h, CM from monocultured and co-cultured MAT-MSCs, MCF7 and MDA-MB231 were collected and analyzed by using the bio-plex multiplex human cytokine and growth factor kits (see Methods). Data were reported as mean ± SD of 5 independent experiments. For multiple comparisons, Kruskall–Wallis test followed by Dunn’s correction was applied. For pairwise comparisons (CM MAT-MSCs + MCF7 vs. CM MAT-MSCs + MDA-MB231) Mann–Whitney test was assessed. * denotes statistically significant values vs. MAT-MSCs (* adjp < 0.05; ** adjp < 0.01); ^§^ denotes statistically significant values compared with MCF7 (^§^ adjp < 0.05; ^§§^ adjp < 0.01); ^#^ denotes statistically significant values vs. MDA-MB231 (^#^ adjp < 0.05; ^##^ adjp < 0.01); ^ç^ denotes statistically significant values compared with MAT-MSCs + MCF7 (^ç^ adjp < 0.05; ^çç^ adjp < 0.01).

Secreted Factor	CM MSC (pg/mL)	CM MCF7 (pg/mL)	CM MDA (pg/mL)	CM MSC + MCF7 (pg/mL)	CM MSC + MDA (pg/mL)
**PDGF**	6.82 ± 0.97	12.06 ± 9.08	7.15 ± 0.95	15.38 ± 8.31	7.65 ± 0.75
**IL-1b**	0.27 ± 0.21	0.14 ± 0.06	0.23 ±0.10	0.22 ± 0.11	0.40 ± 0.16
**IL-1ra**	37.20 ± 10.26	30.83 ± 2.85	32.19 ± 7.67	38.55 ± 4.15 ^§^	51.44 ± 13.96 *^##çç^
**IL-2**	4.56 ± 0.80	3.83 ± 0.99	5.64 ± 0.66	3.72 ± 0.88	5.77 ± 0.47
**IL-4**	0.71 ± 0.26	0.47 ± 0.33	0.71 ± 0.15	0.60 ± 0.22	0.90 ± 0.13
**IL-5**	1.76 ± 0.65	0.66 ± 0.26	1.43 ± 1.02	0.61 ± 0.41	1.29 ± 0.39
**IL-6**	412.40 ± 250.04	2.14 ± 2.57 **	43.20 ± 17.94	421.07 ± 146.91 ^§§^	631.42 ± 286.00 ^##^
**IL-7**	2.00 ± 1.03	3.39 ± 3.29	3.04 ± 0.74	3.51 ± 3.35	2.71 ± 0.19
**IL-8**	4.77 ± 3.91	3.48 ± 2.00	20.45 ± 8.71 *	3.76 ± 2.59	47.00 ± 17.51 **^çç^
**IL-9**	2.33 ± 0.27	4.46 ± 4.95	2.20 ± 0.80	5.23 ± 6.32	2.63 ± 0.36
**IL-10**	5.36 ± 1.47	7.36 ± 2.00	6.78 ± 0.83	7.96 ± 1.9	7.57 ± 1.34
**IL-12**	7.87 ± 3.29	17.45 ± 7.09 *	7.82 ± 3.13	20.10 ± 3.03 **	9.63 ± 3.39 ^çç^
**IL-13**	1.78 ± 0.22	1.40 ± 0.61	1.84 ± 0.30	1.23 ± 0.65	1.81 ± 0.20
**IL-15**	5.10 ± 1.75	4.20 ± 0.97	5.56 ± 1.12	5.32 ± 0.69	7.78 ± 1.88
**IL-17**	5.98 ± 0.68	4.86 ± 1.42	6.96 ± 0.83	5.19 ± 1.68	6.85 ± 0.69
**EOTAXIN**	9.07 ± 3.11	8.19 ± 0.96	8.50 ± 1.23	6.86 ± 4.95	11.54 ± 2.35
**FGF**	12.65 ± 2.23	11.62 ± 4.69	18.36 ± 2.29 *^§^	7.78 ± 5.60	15.09 ± 0.85 ^çç^
**G-CSF**	13.23 ± 1.29	24.70 ± 12.60	20.65 ± 4.94	22.70 ± 8.14	22.73 ± 5.54
**GM-CSF**	19.11 ± 4.30	18.78 ± 7.44	32.69 ± 5.99 *^§^	17.68 ± 6.41	28.28 ± 2.56 ^ç^
**IFN*γ***	15.06 ± 4.90	7.05 ± 4.63 *	8.47 ± 2.63	15.55 ± 3.87 ^§^	17.27 ± 6.19 ^#^
**CXCL10**	14.30 ± 1.53	10.84 ± 5.56	11.41 ± 5.33	9.47 ± 4.97	14.56 ± 1.15
**MCP1**	40.84 ± 28.46	19.02 ± 16.64	18.98 ± 1.66	42.47 ± 18.82	29.24 ± 5.39
**MIP1*α***	0.64 ± 0.27	0.57 ± 0.24	0.92 ± 0.13	0.44 ± 0.30	0.97 ± 0.16
**MIP1*β***	2.39 ± 0.25	2.16 ± 0.61	2.75 ± 0.26	2.81 ± 0.18	2.64 ± 0.15
CCL5	3.47 ± 0.47	3.95 ± 0.97	8.99 ± 3.70 **^§^	4.37 ± 0.31	10.16 ± 4.96 *
**TNF*α***	10.61 ± 4.53	5.94 ± 2.18	8.35 ± 2.52	8.59 ± 3.53	17.02 ± 4.55 ^#ç^
**VEGF**	41.09 ± 15.91	173.44 ± 44.43 *	41.06 ± 9.07 ^§^	231.41 ± 96.53 **	115.12 ± 22.21 *^#^

## Data Availability

The data presented in this study are available on request from the corresponding author.

## Supplementary Materials

The following supporting information can be downloaded at: https://www.mdpi.com/article/10.3390/cancers14215421/s1, Table S1: Primer pairs for qPCR; Table S2: Clinical phenotyping of enrolled population; Figure S1: Heatmap of anthropometric and biochemical data of mammary adipose tissue (MAT) donors; Figure S2: Secretory profile of MAT-MSC isolates and correlation with donor anthropometric/biochemical data; Figure S3: Effect of glucose lowering on MAT-MSC phenotype; Figure S4: Effect of glucose and MAT-MSCs on MCF7 phenotype; Figure S5: Effect of glucose and MAT-MSCs on MDA-MB231 phenotype; Figure S6: Effect of glucose lowering on MCF7 stemness genes.
