# Supplementary material for: Glucose Enhances Pro-Tumorigenic Functions of Mammary Adipose-Derived Mesenchymal Stromal/Stem Cells on Breast Cancer Cell Lines
Source: Cancers (Basel). 2022 Nov 3;14(21):5421. doi: 10.3390/cancers14215421 (PMC9655059; doi:10.3390/cancers14215421)
Supplement: Supplementary file 1 [file cancers-14-05421-s001.zip › cancers-1886925-supplementary.pdf]

GENE	Encoded protein	Forward	T_m (°C)	Reverse	T_m (°C)	Size (bp)
IL8	Interleukin-8 (IL-8)	5'-TGAGAGTGATTGAGAGTGGA-3'	58	5'-TCAAAAACTTCTCCACAACCC-3'	60	275
IL6	Interleukin-6 (IL-6)	5'-CAATGAGGAGACTTGCCTGGT-3'	64	5'-AGCTGCGCAGAATGAGATGA-3'	60	126
VEGF	Vascular Endothelial Growth Factor (VEGF)	5'-TCACAGGTACAGGGATGAGGACAC-3'	74	5'-TCCTGGGCAACTCAGA-3'	50	184
CKDN2A	Cyclin-dependent kinase inhibitor 2A ($p16^{INK4a}$)	5'-CGGAAGGTCCCTCAGACATC-3'	64	5'-AAACTACGAAAGCGGGGTGG-3'	62	138
LMNB1	Lamin B1	5'-GCCCAGATCAAGCTTCGAGA-3'	62	5'-GCTTCCAACTGGGCAATCTG-3'	62	134
ACTA2	Smooth Muscle alpha-Actin (αSMA)	5'-AGAACATGGCATCATCACCA-3'	58	5'-GAGTCATTTTCTCCCGGTTG-3'	60	149
OCT4	Octamer-binding transcription factor 4 (OCT4)	5'-TCAGCCACATCGCCCAGCA-3'	62	5'-AGGGAAAGGGACCGAGGAG-3'	62	242
SOX2	SRY (sex determining region Y)-box 2 (SOX2)	5'-CAACCAGAAAAACAGCCCG-3'	58	5'-CAGCCGCTTAGCCTCGTC-3'	60	196
NANOG	Homeobox protein NANOG	5'-CCTGAAGAAAACTATCCATCC-3'	60	5'-GTTCTGGTCTTCTGTTTCTTG-3'	60	209
PPIA	Peptidylprolyl isomerase A (PPIA)	5'-TACGGGTCCTGGCATCTTGT-3'	62	5'-GGTGATCTTCTTGCTGGTC-3'	58	196

Supplementary Table S1. *Primer pairs for qPCR.*

Parameter	Mean value ± St.Dev.
Age	37.4 ± 13.7
Weight (kg)	73.3 ± 10.7
Height (m)	1.6 ± 0.1
BMI	27.7 ± 3.7
Glucose (mg/dl)	87.1 ± 13.6
Cholesterol (mg/dl)	185.8 ± 31.4
Triglycerides (mg/dl)	90.7 ± 36.9

Supplementary Table S2. *Clinical phenotyping of enrolled population (N=20).*

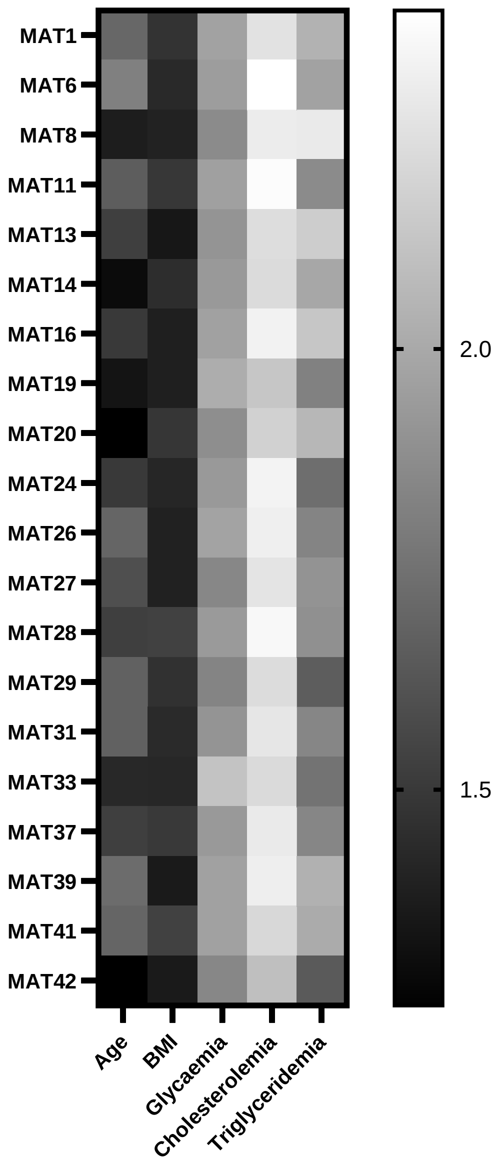

Figure S1 *Heatmap of anthropometric and biochemical data of mammary adipose tissue (MAT) donors*. The log value of each parameter is indicated by the color scale.

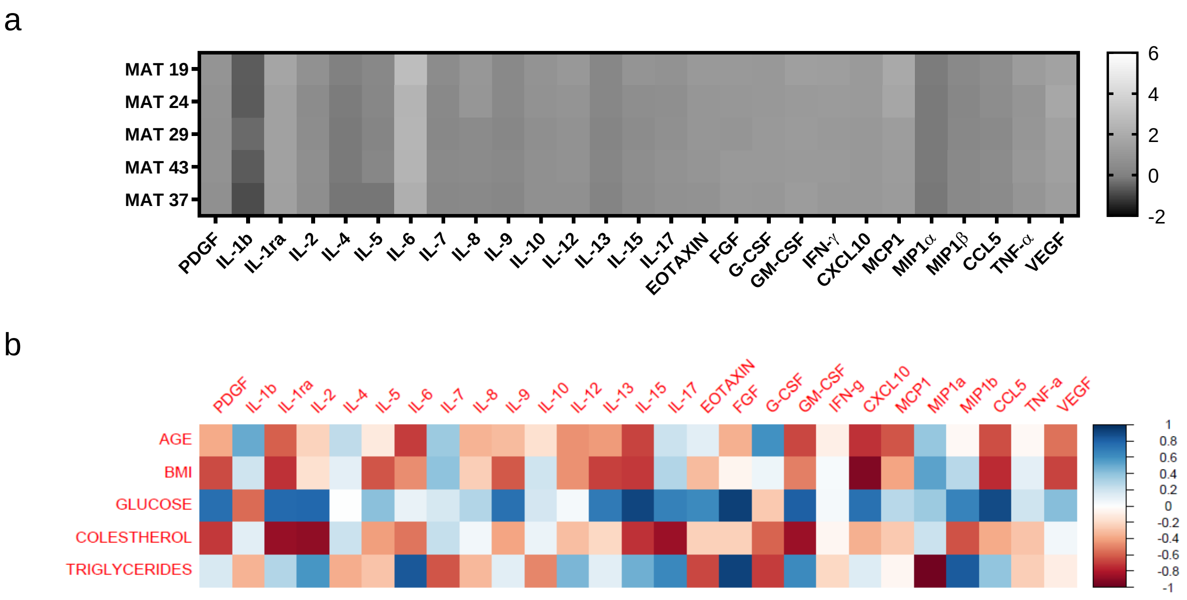

Figure S2 *Secretory profile of MAT-MSC isolates and correlation with donor anthropometric/biochemical data*. **(a)** Heatmap of secretory profile of MAT isolates. The log value of each cytokine concentration is indicated by the color scale. **(b)** Cytokine and donor anthropometric/biochemical data correlation matrix. Correlation matrix was obtained with Pearson Correlation test, using R statistical platform. Blu colors represent positive correlations; red colors negative correlations, according to the legend on the right.

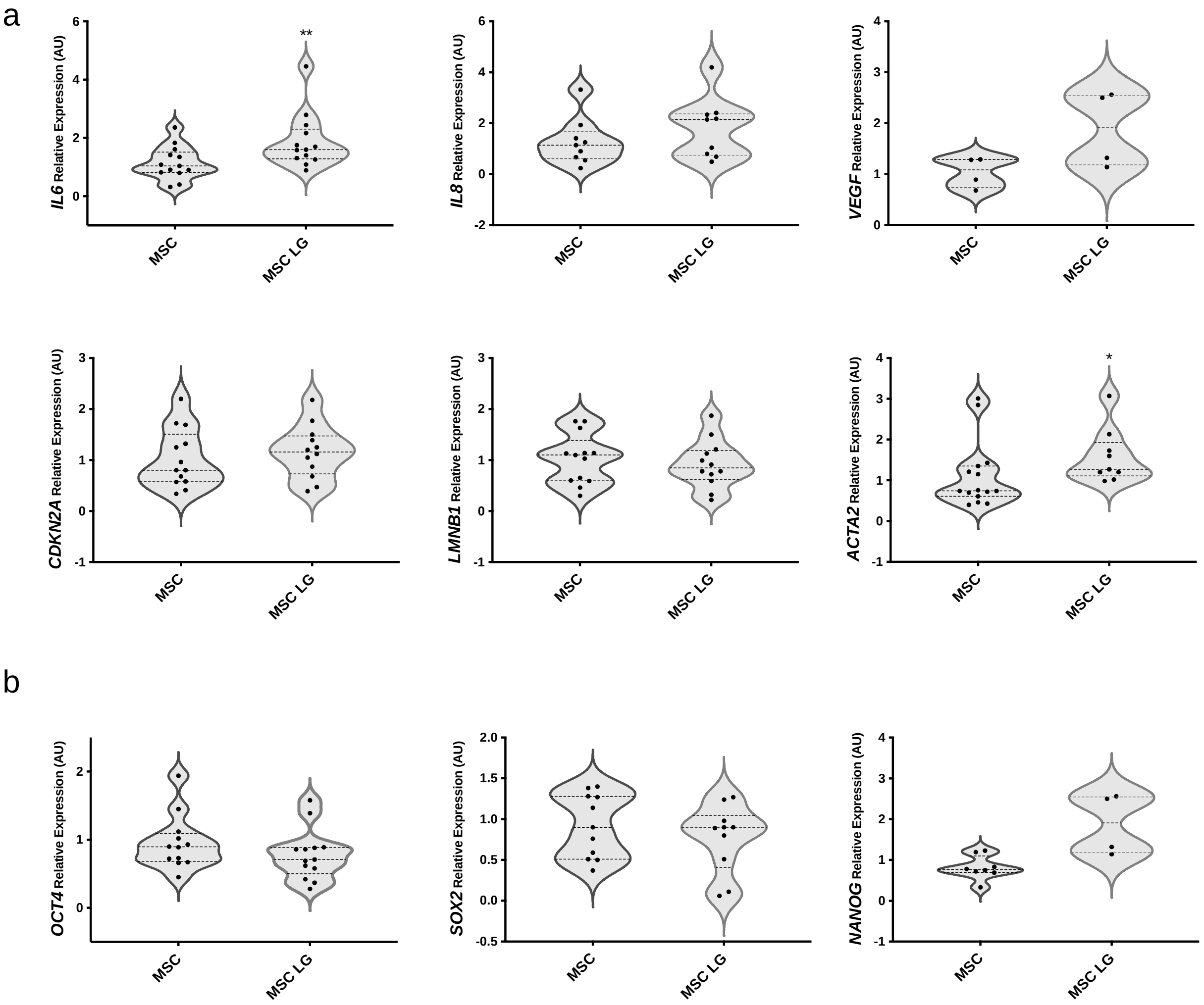

Figure S3 *Effect of glucose lowering on MAT-MSC phenotype.* MAT-MSCs were exposed to 25mM glucose (MSC) or 5.5mM glucose (low glucose- MSC LG) for 72 hours. mRNA expression levels of IL6, IL8, VEGF, CDKND2, LMNB1, ACTA2(a), and of OCT4, SOX2, NANOG were determined by qPCR (see Methods). Data were normalized on Peptidyl-prolyl cis-trans isomerase A (PPIA) gene as internal standard. Results were represented as violin plot of 9-13 independent experiments showing mRNA levels of IL6, IL8, VEGF, CDKND2, LMNB1, ACTA2 (a) and of OCT4, SOX2, NANOG (b) in MSC LG relative to those in MSC. Data were analyzed using the non-parametric Mann Whitney test for pairwise comparisons. * denote statistically significant values compared with MSC (*pval<0.05; **pval<0.01).

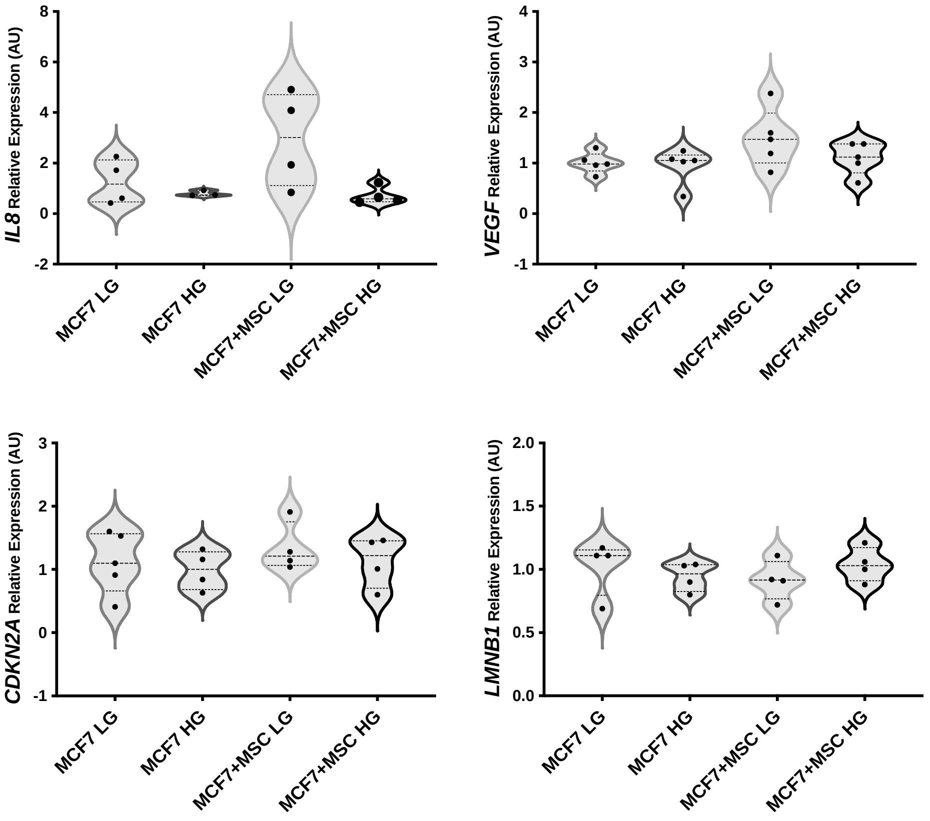

Figure S4 *Effect of glucose and MAT-MSCs on MCF7 phenotype.* MCF7 were co-cultured – and not – with MAT-MSCs in low glucose (5.5 mM; LG) or high glucose (25mM; HG) medium. After 72 hrs, mRNA expression levels of senescence markers (IL8, VEGF, CDKN2A, LMNB1) were determined by qPCR (see Methods and Table 4). Data were normalized on PPIA gene as internal standard. Results were represented as violin plots of 4-5 independent triplicate experiments, showing mRNA levels of IL8, VEGF, CDKN2A, LMNB1 in MCF7 HG, MCF7+MSC LG/HG relative to those in MCF7 LG.

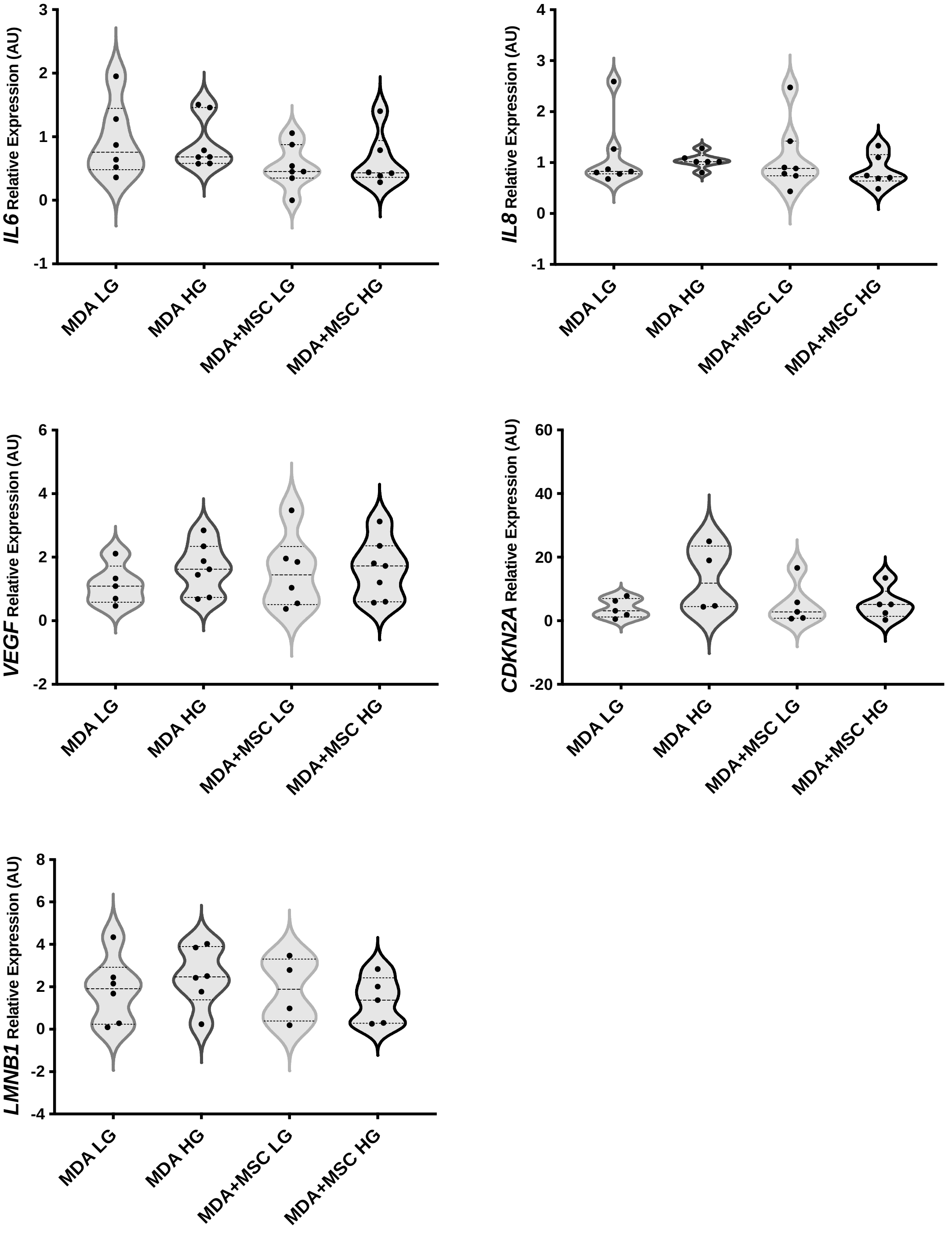

Figure S5 *Effect of glucose and MAT-MSCs on MDA-MB231 phenotype.* MDA-MB231 were co-cultured – and not – with MAT-MSCs in low glucose (5.5 mM; LG) or high glucose (25mM; HG) medium. After 72 hrs, mRNA expression levels of senescence markers (IL6, IL8, VEGF, CDKN2A, LMNB1) were determined by qPCR (see Methods and Table 4). Data were normalized on PPIA gene as internal standard. Results were represented as violin plots of 4-5 independent triplicate experiments, showing mRNA levels of IL6, IL8, VEGF, CDKN2A, LMNB1 in MDA HG, MDA+MSC LG/HG relative to those in MDA LG.

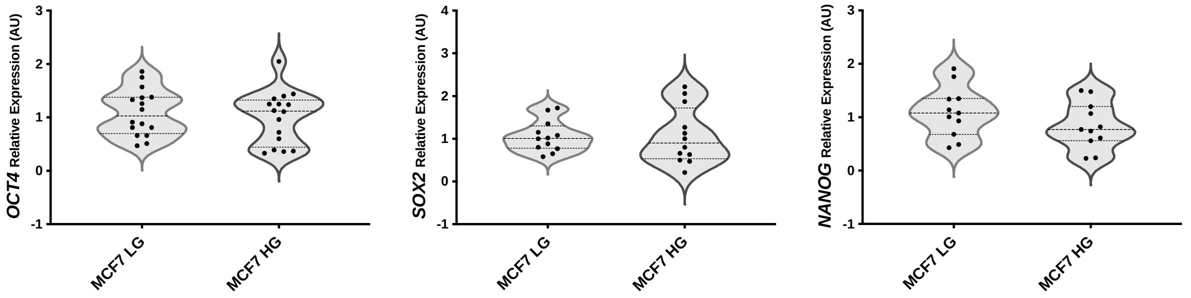

Figure S6 *Effect of glucose lowering on MCF7 stemness genes.* MCF7 were exposed to 25mM glucose (MCF7 LG) or 5.5mM glucose (MCF7 LG) for 72 hours. mRNA expression levels of OCT4, SOX2, NANOG were determined by qPCR (see Methods). Data were normalized on Peptidyl-prolyl cis-trans isomerase A (PPIA) gene as internal standard. Results were represented as violin plot of 9-13 independent experiments showing mRNA levels of OCT4, SOX2, NANOG in MCF7 HG relative to those in LG.

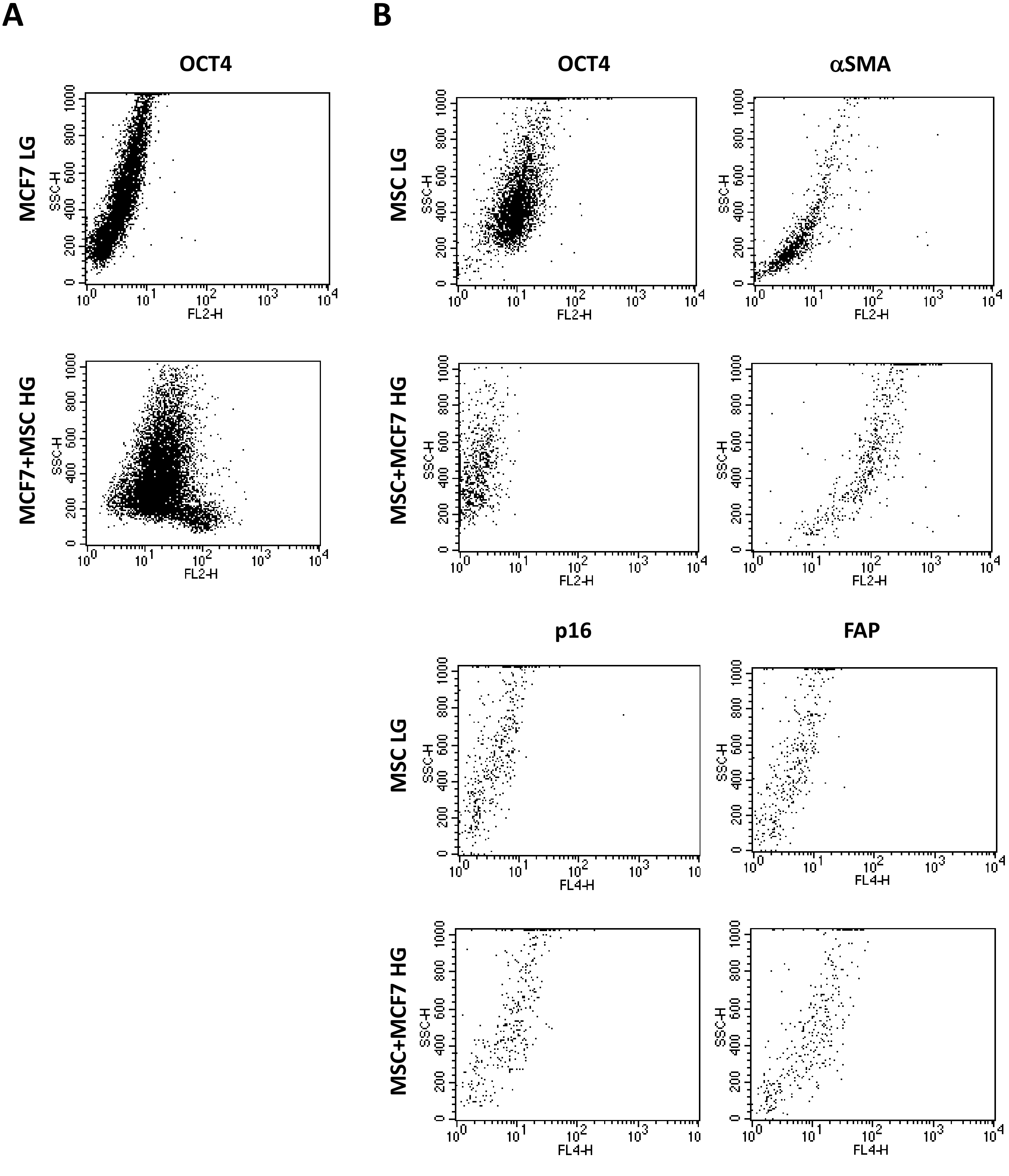

Figure S7 *Cytofluorimetric analysis of MAT-MSCs - MCF7 spheroids.* 3D-cultures were set up by using MCF7 and MAT-MSCs (ratio 4:1) in LG or HG culture media. After 72hrs, spheroids were mechanically disaggregated and cells were stained for cytofluorimetric analysis. Representative dot plots show a) MCF7 stained with PE-anti-OCT4 upon 3D monoculture in LG (control cells) or co-culture with MAT-MSCs in HG, b) MAT-MSCs stained with PE-anti-OCT4, PE-anti-α-SMA, APC-anti-FAP and APC-anti-p16 upon 3D monoculture (control cells) or co-culture with MCF7 in HG.
